# Comparison of a standard computer‐assisted cognitive training program to a music enhanced program: A mixed methods study

**DOI:** 10.1002/cnr2.1325

**Published:** 2020-12-10

**Authors:** Theresa M. Smith, Wanyi Wang

**Affiliations:** ^1^ Texas Woman's University Houston Texas USA

**Keywords:** behavioral science, breast cancer, complementary medicine, survival

## Abstract

**Background:**

Between 17 and 75% of breast cancer survivors (BCS) experience long‐term cognitive deficits such as deficits in memory, attention, processing speed, and executive function.

**Aims:**

This study aimed to (a) compare effects of a standard computer‐assisted cognitive training (CACT) program to a CACT program enhanced with music (CACT+A) to improve focus and concentration on BCS' memory, cognition, quality of life (QOL), and participation in everyday activities; and (b) garner participants' perspectives of effects of the programs to determine best practice.

**Methods:**

An embedded design was employed in this mixed methods study. Participants who reported cognitive problems were recruited through breast cancer support groups. Four pre and post‐tests were used followed by a qualitative interview.

**Results:**

Twenty‐five BCS, ages 31 to 72 years participated. The CACT group demonstrated significantly improved pre to post‐test scores for working memory, QOL, and three subscales of the Model of Human Occupation Screening Tool (MOHOST) measuring participation in everyday activities. The CACT+A group had significant improvement for four FACT‐Cog cognitive function subscales and the total score. Five themes emerged from the interview: *Cognitive skill, Strategy learned, No change, QOL factors,* and *Participation in everyday activities.* The CACT+A group expressed experiencing a larger ratio of improvements, most notably for memory and QOL factors.

**Conclusion:**

CACT+A is an auspicious intervention option for BCS who self‐report cognitive issues. It is convenient to participate in at home and allows BCS to safely self‐isolate if need be.

This study is a registered clinical trial protocol: TexasWU record 19 959.

## 1 INTRODUCTION

Cancer is a continuing disease in our society with greater than 1.8 million new cases projected to be diagnosed in 2020.^1^ In women, about 30% of newly diagnosed cancers will be breast cancers.^2^ As of January 1, 2019, there were 3.8 million women breast cancer survivors (BCS) in the United States.^3^ Researchers have estimated that between 17 and 75% of them experience long‐term cognitive deficits such as deficits in memory, attention, processing speed, and executive function.^4^ Such cognitive deficits of BCS are under diagnosed and can result in decreased participation in everyday activities.^5^, ^6^ Researchers^7^, ^8^, ^9^, ^10^ have shown rehabilitation can improve participation in everyday activities and quality of life (QOL). Rehabilitation generally consists of patients learning compensation skills to replace cognitive skills and/or remediation of cognitive deficits through practice of cognitive skills. Cognitive rehabilitation for remediation of cognitive skills can be provided to BCS with computer‐assisted training.^8^, ^10^, ^11^, ^12^, ^13^

Previous computer‐assisted cognitive training (CACT) programs for BCS focused on improving a variety of cognitive skills such as processing speed,^10^ and executive function including working memory, cognitive flexibility, multitasking, planning, and attention.^8^ Kesler et al^11^ also concentrated on improving executive function and their intervention, consisting of computer exercises using visual stimuli, resulted in significant improvements in cognitive flexibility, verbal fluency, and processing speed, as well as marginal improvement in verbal memory. They conjectured that larger effects may have resulted in their study if a combination of visual and auditory exercises were employed, but auditory exercises were not available at that time. In 2019, the first author and a co‐researcher compared a 4‐week CACT program using primarily visual exercise to one with predominately auditory exercises. Both groups got 10 computer exercises for each exercise session but the primarily visual group was provided more of the visual exercises such as visual attention, visual and spatial memory, and visual memory, and the audio group got more auditory working memory, verbal memory, and verbal and visual memory exercises. The researchers found that CACT with primarily auditory exercises did not result in any greater outcome scores than CACT with primarily vision exercises. Both groups demonstrated improved outcomes for perceived cognitive function and QOL for BCS. There was no control group.^12^

The first author and a co‐researcher have previously established that CACT improves outcomes for perceived cognitive function and QOL for BCS.^12^ Remediation for cognitive deficits is dependent upon the premise that brain neuroplasticity can be achieved^14^; computer brain‐training exercises can facilitate plasticity.^15^ In addition, some types of music can elicit specific brain waves that promote increased focus and concentration.^16^ It is also likely that music interventions can affect brain plasticity due to its shared neural systems for reward, arousal, and affect regulation.^17^

For this study, we were interested in investigating the effects on memory, cognition, QOL, and participation in everyday activities of BCS completing CACT enhanced with music to increase focus and concentration. We contended that in addition to using CACT to improve cognition and QOL in BCS, audio input of music designed to improve focus and concentration tasks should be examined, and if indicated included as a standard of care. The purpose of this study was 2‐fold. First we wanted to compare the effects of a standard CACT program to one with the same computer exercises, but which was enhanced with music to improve focus and concentration (CACT+A) on memory, cognition, QOL, and participation in everyday activities. Second, we wanted to garner participants' perspectives of effects of the two CACT programs to aid in determining best practice when using CACT. We hypothesized that a CACT+A would have greater change scores than a CACT on memory, cognition, QOL, and participation in everyday activities. We sought to answer the research question: “What are the effects of a standard computer‐assisted cognitive training program compared to a computer‐assisted cognitive training program enhanced with music on memory, cognition, quality of life, and participation in everyday activities perceived by breast cancer survivors?”

## 2 METHODS

### 2.1 Research design

A mixed methods study was employed in this study based on the belief that one data set would not be sufficient.^18^ Utilizing this mixed methods allowed authors to combine elements of quantitative and qualitative methods to reach a greater depth of understanding and achieve corroboration of findings.^19^ An embedded design was used with a qualitative strand embedded in the quantitative experiment to provide a secondary role of gaining participant perspectives.^18^ Pre and post‐tests were collected with outcome instruments and then a qualitative interview was performed. The major component was the quantitative data with a qualitative strand embedded after post‐test quantitative data collection to allow understanding of how participants viewed changes and their experiences. Their feedback would be used to improve future interventions.

### 2.2 Participants

A convenience sampling was used to collect the sample. Specifically, participants were recruited from three different breast cancer support groups and through one Facebook group that provided information to BCS. Once the participants were included in the study, they were randomly assigned into one of two groups. A *priori* power analysis was conducted using G*Power 3.1. With a desired level of power set at 0.80, an alpha (α) level at 0.05, and a moderate effect size of 0.30 (f) for repeated measures ANOVA (2 time points × 2 groups), it was determined that a minimum of 24 participants were required to ensure adequate power. The first author visited two of the support groups to present the proposed study and collect contact information of potential participants. A written description of the proposed study was sent to the director of the third support group and to the manager of the Facebook group. Potential participants contacted the first author and were given further information about the study. If they wanted to participate, a time and place for pretesting was arranged. Inclusion criteria for participants were: must be a BCS and self‐report cognitive problems, which they attributed to their breast cancer treatment. Exclusion criteria were persons who could not read or understand spoken English or who self‐reported they had disorders that may affect their cognition including major mental disorder, central nervous system disorders, Alzheimer's disease, dementia, developmental delay, traumatic brain injury, or cerebral accident. Quantitative and qualitative data were collected from all study participants in both intervention groups.

### 2.3 Instruments

Four quantitative and one qualitative outcome measures were employed. To measure working memory, the forward digit span was used. It has been shown to discriminate between BCS and controls.^20^ It takes approximately 10 minutes to administer and has high reliability (0.891) for forward span and low for backwards span (0.598).^21^ A total for digit span and for digit span length were collected. Cognitive function was measured with the 37‐item Functional Assessment of Cancer Therapy‐Cognitive Function (FACT‐Cog).^22^ The FACT‐Cog contains four subscales of: perceived cognitive impairments, comments from others, perceived cognitive abilities (PCA), and impact of cognition on QOL, which are summed to provide a score. All subscales of the FACT‐Cog and the total score were used in the study. The FACT‐Cog subscales of perceived cognitive impairment and comments of others are reverse scored. The FACT‐Cog demonstrates acceptable test‐retest reliability (0.707) and validity (0.762). QOL, as it relates to cancer survival, was measured with the 41 item QOL —Cancer Survivors (QOL‐CS).^23^ Factors affecting QOL for BCS are very different than factors for other conditions. The overall test re‐test reliability of the QOL‐CS is 0.89. The Model of Human Occupation Screening Tool (MOHOST) is an assessment that determines the extent to which motivation, pattern of occupation, communication and interaction skills, process skills, and environment factors facilitate or restrict an individual's participation in everyday activities. We sought to determine whether strategies used to perform CACT would transfer to everyday activities. The MOHOST has shown good construct validity, item separation reliability, and concurrent validity.^24^ We modified the MOHOST for our purposes by omitting the subset of communication and interaction skills as our intervention did not target these skills. Further, we considered the time to perform the pre and post‐tests too short to obtain a valid measure of communication and interaction skills. A higher score on all subscales of all four quantitative outcome measures indicates better functioning.

Qualitative data were collected with a semi‐structured open‐ended question interview. Participants were asked four questions; each question aligned with one of the quantitative outcome measures (see Table 1). Thereby participants were asked to note any changes in their memory, cognition, QOL, and in participation in everyday activities, they attributed to participating in the study.

**TABLE 1:** Qualitative interview

Having completed this study…
Question 1. What, if any, changes in your memory have you noticed?
Question 2. What, if any, changes in your cognition have you noticed?
Question 3. What, if any, changes in your quality of life have you noticed?
Question 4. How have you applied anything you learned in this study to your everyday activities?

### 2.4 Research protocol

The study and consent form were approved through the authors' affiliated university institutional review board conforming to recognized standards of Declaration of Helsinki. Prior to any data collection, participants provided written informed consent at one of two sites based on participants' county of residence. Participants were randomly assigned to either the CACT or the CACT+A group, and administered the quantitative instruments described above which took approximately 45 minutes. Participants were then instructed on how to access and perform the computer‐assisted exercises on the internet. Participants in the CACT+A group were issued headphones and an USB with a publicly available 2 hours and 30 minutes album of music championed to increase focus and concentration. All computer‐assisted training was performed in a location of choice by the participant. Tablets were available if any participants did not have a device to access the Internet, but no one required one. An email was sent to the participants the night before their exercises began with their assigned password and a reminder to do their computer exercises for 30 minutes a day, 5 out of 7 days a week for 1 month. The total time of required computer exercises was 20 hours.

Computer software used for the CACT was from HAPPYneuronPro^25^ which has nine different types of cognitive exercises including auditory, verbal and visual memory, verbal memory, executive functioning, processing speed, spatial memory, visual attention, visual memory, and visual and spatial abilities. Each exercise session was comprised of 10 different exercises run for 3 minutes. When a participant achieved a 100% percent score at a specific level of an exercise, they were given exercises from the next higher level of difficulty. Participants' training was closely monitored to ensure that they completed the required amount of exercises. If they were short training time, they were sent a reminder email. Each week the number of exercises delivered from each type of exercise was modified based upon which cognitive exercise scores most needed improvement. The last week of exercises was selected based upon whatever types of exercises the participant had the lowest scores.

At the end of the month, all pretests were repeated, and the qualitative interview performed in person. The interviews were recorded on a digit recorder and notes about the participants' answers were written on an interview template. Participants who finished the study received $150.00 worth of gift cards as reimbursement.

### 2.5 Data analysis

#### 2.5.1 Quantitative data analysis

Due to the non‐normal distribution of outcome measures, nonparametric analyses were conducted. Wilcoxon signed rank tests were used to examine score changes from pre‐ to post‐test in each group. Mann Whitney *U* tests were conducted to compare group difference at each time point and to compare the change score between the two groups. Spearman's *rho* non‐parametric correlations were then performed to examine the relationships of score changes among the outcomes. All quantitative data were analyzed in SPSS v25. A *P* < .05 was set as significance.

#### 2.5.2 Qualitative data analysis

Qualitative data preparation and analysis began with transcription of the interviews maintaining separate files for the CACT and CACT+A groups. Transcriptions were read for accuracy and to understand the main point of each section,^26^ and then double spaced for initially coding line‐by‐line by hand. Interviews were next uploaded into ATLAS.ti Scientific Software Development GmbH and grounded theory techniques used to analyze data. Using the software, open coding was applied to inductively identify concepts and themes,^26^ and link participants' words to codes frequently using in vivo coding. Constant comparisons were used next in axial coding to group concepts into categories, and theoretical questioning was employed to establish relationships between categories and subcategories.^26^ Selective coding did not occur as it was not the purpose of this article to construct a theory.

Several validation and reliability strategies were used to increase trustworthiness. Creswell and Poth^27^ recommend that at least two means of validation be used. Four validation strategies were used in this study. The first author revealed her bias in noting her previous research. She also had prolonged engagement with the participants and engaged in peer debriefing of the data and research process with a former co‐researcher who is familiar with CACT and the population, BCS. Negative case analysis was demonstrated by indicating when some participants indicated that they did not feel any changes from participation in the study. Reliability of data was enhanced by transcribing the digit recording and reading transcripts for accuracy.^28^ Transcripts were both coded line‐by‐line by hand and then imported into ATLAS.ti for more detailed coding.

## 3 RESULTS

A total of 25 participants (9 in the CACT group, 36% and 16 in the CACT+A group, 64%) were included in the study. Although equal randomization occurred, three of the CACT group were noncompliant with completing their exercises in a timely fashion and had to be dropped from the study. Three other CACT participants dropped out of the study before beginning the exercises due to reportedly being overwhelmed, having technology issues, and no reason stated. Fifty‐six percent of the participants were Caucasian (N = 14), followed by African American (N = 9, 36%) and Asian (N = 1, 4%). There was only one Hispanic participant (N = 1, 4%). The average age of participants was 53.44 ranging from 31 to 72 years old. On average, participants were 4.3 years post‐treatment. However, two were still taking oral chemotherapy.

### 3.1 Quantitative results

#### 3.1.1 Working memory

The total digit span and digit span length were not improved in the CACT+A group, but the digit span length was increased in the CACT group from pretest (*M* = 6.56, SD = .88, Median = 7.00) to post‐test (*M* = 7.33, SD = 1.00, Median = 7.00), *Z* = 2.070, *P* = .038. No significant difference was found between the two groups at either pre or post‐test. Detailed information is displayed in Table 2.

**TABLE 2:** Mean and SD on working memory and cognitive function

Variable	CACT			CACT+A		
	M	SD	Mdn	M	SD	Mdn
Digit span total						
Pre	10.22	1.92	10.00	11.31	2.39	11.00
Post	11.67	2.00	11.00	12.06	2.59	12.00
Change	1.44	2.13	1.00	0.75	1.61	1.00
Length						
Pre	6.56	0.88	7.00	7.50	1.37	7.50
Post	7.33*	1.00	7.00	7.63	1.50	8.00
Change	0.78	0.83	1.00	0.13	1.20	0.00
Perceived cognitive impairment (PCI)						
Pre	41.78	17.46	49.00	39.94	15.88	39.50
Post	42.33	6.93	42.00	50.88*	13.29	54.50
Change	0.56	15.55	−5.00	10.94	12.56	8.00
Comments from others						
Pre	14.67	2.18	16.00	12.75	3.36	14.00
Post	14.11	2.62	15.00	15.06*	1.34	16.00
Change	−0.56	1.81	0.00	2.31**	2.65	2.00
Perceived cognitive ability (PCA)						
Pre	15.33	5.43	16.00	13.06	5.40	12.00
Post	14.44	3.54	13.00	19.00* ^,^ **	5.14	20.50
Change	−0.89	5.28	−1.00	5.94**	6.90	6.00
Impact of cognition on quality of life						
Pre	8.67	3.94	8.00	9.94	3.59	11.00
Post	10.78*	3.90	11.00	11.75*	3.70	12.00
Change	2.11	2.47	2.00	1.81	3.23	2.50
Total cognitive function						
Pre	77.11	23.53	84.00	75.88	24.37	76.50
Post	85.00	17.46	82.00	96.69*	18.76	101.00
Change	7.89	19.09	−3.00	20.81	20.68	18.00
Quality of life (QOL‐CS))						
Pre	6.10	1.35	6.73	5.80	1.37	5.86
Post	6.71*	1.08	6.26	6.07	1.53	5.95
Change	0.61	0.71	0.83	0.27	0.65	0.21

*Note*: *N* = 9 in CACT group, and *N* = 16 in CACT+A group.

Abbreviations: CACT, computer‐assisted cognitive training; CACT+A, computer‐assisted cognitive training enhanced with music; PCA, perceived cognitive ability; PCI, perceived cognitive impairment; M, mean; Mdn, median; QOL‐CS, quality of life; SD, standard deviation.

* *P* < .05 significant different from pretest.

** *P* < .05 significant different from standard computer‐assisted cognitive training group.

#### 3.1.2 Cognitive function

The FACT‐Cog cognitive function subscales include perceived cognitive impairment (PCI), comments from others, PCA, and impact of cognition on QOL. As shown in Table 2, the subscale scores for PCI were significantly increased from pretest (*M* = 39.94, SD = 15.88, Median = 39.50) to posttest (*M* = 50.88, SD = 13.29, Median = 54.50) in the CACT+A group, *Z* = 2.785, *P* = .005. The subscale scores of comments from others were also significantly higher at posttest (*M* = 15.06, SD = 1.34, Median = 16.00) than that at pretest (*M* = 12.75, SD = 3.36, Median = 14.00) in the CACT+A group, *Z* = 2.679, *P* = .007. CACT+A intervention also increased subscale scores for the PCA from pretest (*M* = 13.06, SD = 5.40, Median = 12.00) to post‐test (*M* = 19.00, SD = 5.14, Median = 20.50), *Z* = 2.728, *P* = .006. However, the subscale scores for the above three measures were not impacted in the CACT group. It is worth noting that the subscale scores in the PCA were higher in the CACT+A group as compared to the CACT group at posttest, *U* = 34.000, *Z* = 2.158, *P* = .032. In addition, when the change scores were calculated from pre to posttest, the CACT+A group demonstrated better changes on the subscale score for comments from others (*U* = 26.000, *Z* = 2.672, *P* = .008) and PCA (*U* = 33.500, *Z* = 2.183, *P* = .027) than the CACT group. Taken together from the above results, participants reported improved cognitive function after receiving CACT+A intervention. Regarding the impact of cognition on QOL, the subscale scores were elevated significantly from pre‐ to post‐test in both groups (*p*s < .05), but no significant difference was observed between the two groups. Therefore, the FACT‐Cog total score for cognitive function from the above four subscale scores was significantly improved in the CACT+A group (*Z* = 3.026, *P* = .002), whereas this improvement was not found in the CACT group.

#### 3.1.3 Quality of life

Interestingly, the scores for QOL‐CS were better at post‐test (*M* = 6.71, SD = 1.08, Median = 6.26) than that at pretest (*M* = 6.10, SD = 1.35, Median = 6.73) in the CACT group only, *Z* = 2.253, *P* = .024. The QOL‐CS in the CACT+A group showed marginal improvement at posttest but did not reach statistical significance, *Z* = 1.655, *P* = .098. Therefore, CACT+A did not appear to affect QOL, as it relates to cancer survival from pre‐ to post‐test.

#### 3.1.4 Participation in everyday activities

As seen in Table 3, MOHOST subscale scores for process skills were significantly increased from pre to posttest in both groups (*p*s < .05), but there was not significant difference between the two groups. For the MOHOST subscale scores for pattern of occupation and environment factors, the change scores were significantly lower in the CACT+A group as compared to the CACT group, suggesting that the CACT+A treatment did not improve participation in everyday activities.

**TABLE 3:** Mean and SD on the MOHOST

Variable	CACT			CACT+A		
	M	SD	Mdn	M	SD	Mdn
Motivation						
Pre	14.11	2.26	15.00	14.44	1.71	15.00
Post	14.89	0.93	15.00	14.88	1.20	15.00
Change	0.78	2.59	0.00	0.44	2.10	0.00
Pattern						
Pre	12.78	1.20	13.00	14.75**	1.44	15.50
Post	15.44*	0.88	16.00	14.56	1.59	15.00
Change	2.67	1.32	3.00	−0.19**	1.68	0.00
Process						
Pre	13	1.41	13.00	13.25	1.98	14.00
Post	14.89*	0.93	15.00	14.56*	1.67	15.00
Change	1.89	1.96	2.00	1.31	2.09	1.00
Environment						
Pre	13.33	1.80	14.00	13.94	1.73	14.00
Post	14.67*	1.00	15.00	13.63	1.67	13.50
Change	1.33	1.50	1.00	−0.31**	1.70	0.00

*Note*: *N* = 9 in CACT group, and *N* = 16 in CACT+A group.

Abbreviations: CACT, standard computer‐assisted cognitive training; CACT+A, computer‐assisted cognitive training enhanced with music; M, mean; Mdn, median; SD, standard deviation.

* *P* < .05 significant different from pretest.

** *P* < .05 significant different from comparison group.

#### 3.1.5 Relationship of change scores among FACT‐Cog total score of cognitive function, all FACT‐Cog subscale scores, and QOL‐CS

Lastly, the change scores from pretest to posttest were calculated, and the relationship of change scores between the four subscales of cognitive function and the FACT‐Cog total score were examined on the combined samples. The results, seen in Table 4, show strong relationships between FACT‐Cog total score of cognitive function and three subscale scores, including PCI, comments from others, and PCA (*r*s = 0.627‐0.830). Results indicate better overall cognitive function was associated with higher PCI, comments from others, and PCA. Moreover, PCI, comments from others, and PCA were moderately highly related to each other (*r*s = 0.460‐0.762). However, subscale score of impact of cognition on QOL was not significantly related to any other subscale scores or the FACT‐Cog total score of cognitive function. When it comes to the total QOL‐CS score, it was only positively related to the FACT‐Cog total score of cognitive function (*r* = 0.418).

**TABLE 4:** Spearman's Rho correlations among change scores on cognitive function and quality of life

Variable	Impact of cognition on QOL	Comments from others	PCA	Total perceived cognitive function	Total QOL
PCI	0.196	0.460*	0.762**	0.830**	0.392
Impact of cognition on QOL		0.201	0.180	0.179	−0.166
Comments from others			0.498*	0.627**	0.104
PCA				0.732**	0.256
Total perceived cognitive function					0.418*

Abbreviations: PCA, perceived cognitive ability; PCI, perceived cognitive impairment; QOL, quality of life.

* *P* < .05.

** *P* < .01.

### 3.2 Qualitative results

Five themes and their subthemes emerged from the data and generally reflected the focus of the questions (see Table 5). Themes were *Cognitive skill, Strategy learned, No change, QOL factors,* and *Participation in everyday activities*. Subthemes such as *improved attention*, a subtheme of *Cognitive skill* was linked to a comment from participant 110. She said, “I think the more I pay attention, the more I remember.” One subtheme of QOL *factors* was *boosted confidence*. This subtheme emerged from quotes such as the one from participant 104 when she remarked, “So going through some of these exercises where I was able to recall just helped boost my confidence.” Participants mentioned using different *strategies,* a subtheme of *Strategy learned*, such as visualizing the placement of words, or grouping like objects, slowing down, and practicing math by adding and subtracting license plate numbers.

**TABLE 5:** Effects of computer‐assisted cognitive training programs by interview question

Question #	Themes	Subthemes	Frequency
			CACT|CACT+A
1	Cognitive skill		
		Improved attention	1|4
		Memory improved	2|11
		Thinking clearly	0|4
	Strategy learned		
		Strategies	0|4
	Quality of life factor		
		Calmer	0|2
	No change		3|1
	Total frequency		6|26
2	Cognitive skill		
		Memory improved	2|5
		Better with numbers	0|3
		Processing speed improved	0|2
		Improved attention	7|8
	Strategy learned		
		Strategies	2|4
	Quality of life factor		
		Gained new perspective	0|2
	Total frequency		11|24
3	Cognitive skill		
		Memory improved	0|3
		Improved attention	0|2
	Quality of life factor		
		Boosted confidence	3|4
		Calmer	0|3
		Eats more healthy	0|2
		Gained new perspective	0|2
	Occupational participation		
		Continuing exercises	0|4
	No change		3|4
	Total frequency		6|24
4	Cognitive skill		
		Memory improved	0|7
		Had to be dedicated to study	0|4
		Improved attention	6|5
		Time management better	0|2
	Strategy learned		
		Strategies	8|10
	Quality of life factors		
		Boosted confidence	3|3
		Gained new perspective	2|2
	Participation in everyday activity		
		Continuing exercises	1|5
	Total frequency		20|38
	Grand total		43/112

Abbreviations: CACT, standard computer‐assisted cognitive training; CACT+A, computer‐assisted cognitive training enhanced with music.

To analyze the qualitative data with the quantitative data, qualitative data were transformed into quantitative data, or quantized.^29^ Code counting was the method used in quantizing the qualitative data (see Table 5). In response to question 1 as to changes in memory, content analysis revealed the subtheme of *improved memory* had a ratio of 2:11 comments with a larger ratio of CACT+A reporting improved memory. The total comments of improvement in themes for question 1 were 3:25 with the larger ratio for CACT+A participants. Of note, the ratio of the theme of *No change* yielded a ratio of 3:1 with the larger number ratio of CACT participants reporting *No change*. Qualitative responses for question 2 of changes noted for the theme of *Cognitive skill* resulted in a ratio of 9:18 with the larger ratio for CACT+A participants with improved cognitive skills. Question 3 as to changes in QOL yielded for the theme QOL *factors* a ratio of 3:11 with the CACT+A commenting on three other ways their QOL improved. Total ratio of subthemes was 3:20 with the larger ratio for CACT+A participants commenting on improved QOL. In addition, of note is the ratio of participants reporting *No change* of 3:4 with the larger ratio for CACT+A. Question 4 as to changes in participation in everyday activities yielded only one named everyday activity with a ratio of 1:5 with the larger ratio for CACT+A. Total noted improvements for Question 4 themes/subthemes were 20:38. The grand total of themes/subthemes for all questions was 43:112 with the larger ratio for CACT+A.

## 4 DISCUSSION

Our hypothesis that a CACT+A would have higher improved change scores than a CACT on memory, cognition, QOL, and participation in everyday activities proved only partially correct. The CACT group demonstrated significantly improved pre‐ to post‐test scores for working memory for digit span length, QOL, and three MOHOST subscales of pattern, process, and environment. However, CACT+A did have statistically significant improvement on the FACT‐Cog total score and all four FACT‐Cog subscales scores including the impact of cognition on QOL. The significantly increased score for the FACT‐Cog subscale of impact of cognition on QOL vs only an increase on the QOL‐CS may be indicative that factors other than cognitive deficits contribute to QOL for BCS. The MOHOST processing subscale score was also significantly improved for the CACT+A group which correlates with improved cognitive skills. Given the fact that the groups had the same computer exercise training, it is possible that using the external headphones may have resulted in decreased motivation and environment for participation in everyday activities, as well as an insignificant increase in QOL for the CACT+A group.

Regarding our second aim of the study regarding best practice, we recommend not using headphones in future CACT+A. This determination was based on the answer to our research question on effects of the CACT and CACT+A programs for BCS. There were differences for the groups and with only nine members in the CACT group and 16 in the CACT+A, more changes would be expected for the CACT+A group. It was then surprising that although quantitative findings demonstrated significant improvements for the CACT in memory and QOL, the number of improvements noted from the qualitative data for improved memory (question 1) and QOL (question 3) for the CACT+A group far exceeded those of the CACT group, all things equal. A subtheme only found for the CACT+A group of *had to be dedicated to the study* (question 4), can be interpreted as the use of headphones and the planning it involved adversely affected their participation in everyday activities overall.

The computer‐assisted cognitive exercises in our study did include those to improve working memory but all exercises were run for only up to 3 minutes per day. The primary author and colleague did not previously show improvement in working memory with CACT^12^ and nor did Kesler and colleagues.^11^ The authors have no plausible reason that the CACT significantly improved in digit span length but the CACT+A group did not.

Prior researchers have conducted CACT programs with BCS and we sought to improve upon their programs. A major difference in our program is it is only 4 weeks in length. The CACT program that Kesler et al utilized consisted of five computer exercises completed for 20‐30 minutes, four times a week, for 12 weeks.^11^ Von Ah et al compared an intervention of memory training to a CACT program for speed of processing.^10^ Both interventions were delivered in 1‐hour groups of 3‐5 BCS over 6‐8 weeks. Bray et al evaluated a home‐based CACT intervention that ran for 15 weeks with four training sessions of 40 minutes a week to a group receiving standard of care.^8^ Despite the significant decrease in length of this CACT+A program as compared to previous programs, it resulted in increased cognitive function.

We used some of the same outcome measures as other researchers to best compare study outcomes. For example, Bray et al used the FACT‐Cog and Von et al the FACT‐Cog and the QOL‐CS.^8^, ^10^ Bray et al did measure QOL and Kesler et al subjective cognitive function, but they used different self‐report measures.^8^, ^11^ Kesler et al used the digit span as we did to measure working memory with similar results noted above.^11^

There were several limitations of this study. Participants were selected based on self‐report of cognitive deficits secondary to cancer treatment and not by an objective measure. Participants were not excluded from the study if they were currently receiving cancer treatment and their current treatment may have adversely affected their study outcomes. Some participants required more reminders to complete their exercises in a timely fashion, and the first author did remind them. The MOHOST had been adapted from its standard form and it is generally completed while observing a patient. The only observation made during the pretest was on how the participant completed the pretests. The only observation scored for the MOHOST posttest was how the participant did on the computer exercises at home. CACT+A may have had more issues affecting MOHOST subscale scores secondary to greater requirements needed to use the audio equipment. Further, the first author was responsible in scoring over 95% of the MOHOST pre and post tests and felt her scoring was very subjective. There are no interrater reliability studies on the MOHOST. In addition, the primary author did not inquire as to when or how long CACT+A group listened to the music on the issued USB or other music. It is also plausible that listening to the music may have interfered with computer training. Lastly, qualitative data were collected with a short questionnaire and this may have led to limited depth of content being obtained.

Study findings suggest implications for practice and research. Participants need detailed instructions on how to use the software including how they can monitor their own progress and participation in the exercises. Practice implications are that CACT+A can be used as a home program in conjunction with usual treatment. A therapist can select appropriate cognitive skills upon which they reason the patient needs improvement and monitor a patient's progress and participation asynchronistically. Future research should include education on how to apply strategies utilized in the CACT+A to improving participation in everyday activities. Assessment for factors other than cognitive deficits that may affect QOL of BCS should be employed and deficits treated accordingly. Another means of providing audio input should be considered to reduce patient burden of needing additional equipment and a location to accommodate the equipment. An observational assessment for working memory and/or cognitive skills should be considered.

Study findings suggest several implications for future research. A control group should be included as well as larger group sizes, and more time points of assessment to determine retention of intervention effects. An observational measure of memory and cognitive skills would be more objective. Improvements to the CACT+A should include adding assessments and interventions addressing other factors adversely affecting QOL for BCS. A quantitative measure is needed to measure changes in participation in everyday activities. A different mixed methods design and/or a different means of collecting qualitative data could be employed to more evenly utilize both quantitative and qualitative data. Given the COVID‐19 pandemic, CACT+A should occur totally online.

## 5 CONCLUSION

By using an embedded design in this mixed methods study, we were able to report results of quantitative outcomes measures and provide participants' perspectives on their performance in the study with the qualitative interview results. Notwithstanding study limitations, BCS who had the CACT+A program self‐reported significantly improved perceived cognitive function and expressed experiencing greater ratios of improvements for memory and QOL. CACT+A is an auspicious intervention option for BCS who self‐report cognitive issues. It is convenient to participate in at home and allows BCS to safely self‐isolate if need be.

## CONFLICT OF INTEREST

The authors have no commercial or proprietary interests in HAPPYneuronPro products or company. Nor do they have any other conflicts of interests.

## AUTHOR CONTRIBUTIONS

Both authors had full access to the data in the study and take responsibility for the integrity of the data and the accuracy of the data analysis. *Conceptualization*, Theresa M. Smith and Wanyi Wang; *Methodology*, Theresa M. Smith and Wanyi Wang; *Investigation*, Theresa M. Smith; *Formal Analysis*, Theresa M. Smith and Wanyi Wang; *Resources*, Theresa M. Smith and Wanyi Wang; *Writing—Original Draft*, Theresa M. Smith; *Writing—Review & Editing*, Theresa M. Smith and Wanyi Wang; *Visualization*, Wanyi Wang; *Supervision*, Theresa M. Smith; *Funding Acquisition*, Theresa M. Smith.

## ETHICS STATEMENT

The affiliated IRB of the authors approved this study and the consent form conforming with recognized standards of Declaration of Helsinki and assigned the following reference number: 1959.

## Data Availability

The data that support the findings of this study are available from the corresponding author upon reasonable request.
